# Efficacy and Safety of Anti-PD1/PDL1 in Advanced Biliary Tract Cancer: A Systematic Review and Meta-Analysis

**DOI:** 10.3389/fimmu.2022.801909

**Published:** 2022-03-02

**Authors:** Qi Jiang, Jinsheng Huang, Bei Zhang, Xujia Li, Xiuxing Chen, Bokang Cui, Shengping Li, Guifang Guo

**Affiliations:** ^1^ VIP Department, Sun Yat-sen University Cancer Center, Guangzhou, China; ^2^ State Key Laboratory of Oncology in South China, Sun Yat-sen University Cancer Center, Guangzhou, China; ^3^ Collaborative Innovation Center for Cancer Medicine, Sun Yat-sen University Cancer Center, Guangzhou, China; ^4^ Guangdong Provincial Key Laboratory of Malignant Tumor Epigenetics and Gene Regulation, Department of Medical Oncology, Sun Yat-sen Memorial Hospital, Sun Yat-sen University, Guangzhou, China; ^5^ Department of Pancreaticobiliary Surgery, Sun Yat-sen University Cancer Center, Guangzhou, China

**Keywords:** biliary tract cancer (BTC), anti-PD1, anti-PDL1, anti-CTLA4, antiangiogenesis, chemotherapy, meta-analysis

## Abstract

**Background:**

Anti-programmed cell death protein 1 and its ligand (anti-PD1/PDL1) have been proposed as a promising therapeutic option for advanced biliary tract cancer (aBTC). Given the scarce quantitative analyses of anti-PD1/PDL1 in aBTC, we thus did a meta-analysis to assess the benefits and risks of this emerging treatment strategy in patients with aBTC.

**Methods:**

PubMed, Embase, the Cochrane Library, Web of Science, and meeting resources were searched for relevant studies. The main endpoints were median progression-free survival (mPFS), median overall survival (mOS), objective response rate (ORR), disease control rate (DCR), any-grade adverse events (AEs), and grade 3–4 AEs.

**Results:**

Twenty-eight studies with 1,338 participants were included. The best curative effect was found in the anti-PD1/PDL1 combined with anti-CTLA4 and chemotherapy group (mPFS: 12.4 months; mOS: 16.0 months; ORR: 45.1%; DCR: 95.0%), followed by the anti-PD1/PDL1 plus chemotherapy group (mPFS: 8.2 months; mOS: 14.8 months; ORR: 36.3%; DCR: 84.6%), the anti-PD1/PDL1 plus antiangiogenesis group (mPFS: 4.9 months; mOS: 10.2 months; ORR: 17.5%; DCR: 68.7%), the anti-PD1/PDL1 plus anti-cytotoxic T lymphocyte antigen 4 (anti-CTLA4) group (mPFS: 2.9 months; mOS: 8.3 months; ORR: 9.9%; DCR: 36.8%), and the anti-PD1/PDL1 monotherapy group (mPFS: 2.5 months; mOS: 7.6 months; ORR: 6.8%; DCR: 34.7%). Compared with anti-PD1-containing regimens, anti-PDL1-containing regimens achieved preferable mPFS (11.1 vs. 3.8 months), mOS (12.2 vs. 9.8 months), and ORR (23.7% vs. 17.4%), despite a similar DCR (61.1% vs. 61.3%). The mPFS, mOS, ORR, and DCR were 10.6 months, 15.8 months, 42.3%, and 88.6% of first-line anti-PD1/PDL1 and 3.0 months, 9.1 months, 11.6%, and 51.1% of second-line therapy or beyond, respectively. There were 80.6% and 34.0% of the patients suffering any-grade AEs and grade 3–4 AEs. Anti-PD1/PDL1 monotherapy might be considered as a safer alternative than combination regimens. Meanwhile, obvious toxicities in the first-line setting could not be neglected.

**Conclusions:**

Anti-PD1/PDL1 showed encouraging efficacy and acceptable safety profile in aBTC and, thus, could be an alternative treatment.

## Introduction

Biliary tract cancer (BTC), including intrahepatic cholangiocarcinoma (ICC), extrahepatic cholangiocarcinoma, and gallbladder cancer, is a heterogeneous group of malignant tumors that arises from the epithelium of the bile duct or gallbladder. The incidence of BTC, which accounts for roughly 10%–15% of hepatobiliary malignancies, is increasing progressively worldwide (1–3). Unfortunately, BTC carries a poor prognosis with a 5-year survival rate between 5% and 18%. Diagnosing BTC at an early stage remains elusive given its insidious onset and strong invasion, which poses a barrier to prompt surgical intervention, the only potentially curative treatment for BTC (4). Even for patients suitable for surgery, radical resection rate is still low and relapse rate cannot be ignored (5). Accordingly, palliative chemotherapy remains the mainstay of treatment for the majority of patients suffering BTC. The ABC-02 and ABC-06 studies demonstrated the antitumor effects of gemcitabine plus cisplatin (GemCis) and modified fluorouracil plus oxaliplatin (mFOLFOX), respectively, which established GemCis as first-line therapy and mFOLFOX as second-line therapy (6, 7). Notwithstanding the above, the exact benefits of the recognized chemotherapy regimens are still dismal. Furthermore, beyond the second line, no standard chemotherapy regimen has emerged.

Immune checkpoint inhibitor (ICI) has the power to restore T-cell-mediated tumor cell killing and deplete regulatory T cells (Treg) by blocking immune checkpoint molecules like programmed cell death protein 1 (PD1), programmed cell death ligand 1 (PDL1), and cytotoxic T lymphocyte antigen 4 (CTLA4) (8, 9). This ability has earned extensive interest from researchers. The past decade has yielded tremendous insights into the antitumor activity of PD1/PDL1 antibodies, which has scored marvelous achievements in a range of solid tumors such as melanoma, non-small cell lung cancer, renal cell carcinoma, bladder cancer, and Hodgkin’s lymphoma (10–14). Upregulation of PD1 or PDL1 has been observed in BTC tumor tissues, justifying the use of anti-PD1/PDL1 in BTC (15–17). On the other hand, considerable attention has also been paid to the limited objective response rate (ORR) and acquired resistance of anti-PD1/PDL1 monotherapy (18, 19). That is why it is desirable to exploit efficient combination regimens with PD1/PDL1 inhibitors for BTC.

The addition of anti-CTLA4 to anti-PD1/PDL1 may have an enhanced efficacy on T-cell-mediated antitumor responses through non-redundant immune checkpoint blockade (20). The clinical benefits of this combination have been demonstrated in melanoma, renal cell carcinoma, and colorectal cancer (21–23). Meanwhile, the combination of anti-PD1/PDL1 and antiangiogenesis is another treatment regimen worth looking forward to. Apart from overexpression of vascular endothelial growth factor found in 53% ICC, antiangiogenic therapy also has synergistic effects with anti-PD1/PDL1 in the treatment of cancer through reducing Treg and immunosuppressive cytokines as well as converting the complex tumor microenvironment (24–27). Anti-PDL1 plus bevacizumab has shown amazing efficacy for hepatocellular carcinoma in the IMbrave150 study (28). What is more, conventional chemotherapy may enhance both innate and adaptive immunity and help recover immunosurveillance, supporting the rationale of using anti-PD1/PDL1 combined with chemotherapy (29, 30).

Herein, we did a meta-analysis for the following purposes: 1) to delineate the role of anti-PD1/PDL1 in advanced biliary tract cancer (aBTC), either as monotherapy or in combination with other therapies; 2) to make a comparison between anti-PD1 and anti-PDL1; and 3) to figure out the differences between first-line therapy and second-line therapy or beyond.

## Materials and Methods

This meta-analysis was performed in accordance with the Preferred Reporting Items for Systematic Reviews and Meta-Analyses (PRISMA) guidelines. This study was not registered.

### Search Strategy

We systematically retrieved literature published from database inception up until May 7, 2021, by searching PubMed, Embase, the Cochrane Library, and Web of Science. There were no limitations on language, region, age, and duration of follow-up. We searched the following combined Medical Subject Headings (MeSH) terms and text word: “Biliary Tract Cancers,” “Cholangiocarcinomas,” “Gallbladder Cancers,” “PD1,” and “PDL1.” The search strategy used for PubMed is available in **Supplementary File 1**. In addition, reference lists of reviews and meeting resources (including abstracts and posters) of the American Society of Clinical Oncology (ASCO) and European Society of Medicine Oncology (ESMO) until September 30, 2021, were also scanned through manual search.

### Selection Criteria

The inclusion criteria were as follows: 1) prospective or retrospective clinical studies; 2) patients diagnosed with aBTC and treated with anti-PD1/PDL1, either as monotherapy or combined with antiangiogenensis, anti-CTLA4, or chemotherapy; and 3) studies reporting any of the following outcomes: progression-free survival (PFS), overall survival (OS), ORR, disease control rate (DCR), any-grade adverse events (AEs), and grade 3–4 AEs.

The exclusion criteria were as follows: 1) editorials, letters, reviews, and case reports; 2) cell or animal experiments; 3) anti-PD1/PDL1 combined with drugs other than antiangiogenensis, anti-CTLA4, or chemotherapeutic agents; 4) no results provided or outcomes not relevant; and 5) duplicate studies.

### Quality Assessment

Thirteen studies consisted of 11 prospective studies (31–41) and 2 retrospective studies (42, 43), which were assessed by the Risk Of Bias In Non-randomized Studies of Interventions (ROBINS-I) (44) and the JBI critical appraisal tool for case series (45), respectively. There were 14 studies [1 randomized study (46), 2 non-randomized comparative studies (47, 48), 10 single-arm studies (49–58), and 1 retrospective study (59)] with no full text available, which is why we gave up the corresponding quality assessments. What is more, we also did not assess the quality of one study reporting safety run-in results of a randomized, two-arm, non-comparative trial (60) due to the paucity of validated evaluation tools designed for this kind of trial.

### Data Extraction

Data extraction was performed independently by two investigators (QJ and XL) whose disagreements would be settled by further discussion with a third investigator (GG). The following information from each study was recorded: first author, publication year, region, study type, median follow-up, disease status, drug, clinical setting, line of therapy, sample size, median age, gender, efficacy outcomes [including median progression-free survival (mPFS), 6-month PFS and 12-month PFS, median overall survival (mOS), 6-month OS, 12-month OS, ORR, DCR, complete response (CR), partial response (PR), stable disease (SD)], and safety outcomes (including any-grade AEs and grade 3–4 AEs). We used the package digitize of software R version 3.6.3 for obtaining survival data from the Kaplan–Meier curves (K-M curves). The number at risk, number censored, and number of events were estimated based on the method proposed by Tierney et al. (61).

### Statistical Analysis

The pooled estimates of ORR, DCR, CR, PR, SD, any-grade AEs, and grade 3–4 AEs were calculated using STATA SE version 15. Besides subgroup analyses, we also provided pooled results after omitting studies that may be the source of heterogeneity. Heterogeneity across studies was evaluated by the Cochran *Q* chi-square test and *I* ^2^ statistic, with *P <*0.1 for the *Q* test deemed to have high heterogeneity and *I* ^2^ >50% regarded as an indicator of moderate-to-high heterogeneity. If separate verdicts from the *Q* test and *I* ^2^ statistic were at opposite poles, we would give priority to the conclusion from the *I* ^2^ statistic since the former is proverbially underpowered to detect heterogeneity (62). The robustness of the results was checked by sensitivity analyses. Funnel plots were drawn to evaluate publication bias. Moreover, Egger’s test was used to assess funnel plot asymmetry and *P <*0.1 indicated significant publication bias. Of note, the sensitivity of Egger’s test decreases when the number of included data was smaller than 20 (63), in which case we did not perform Egger’s test. Differences between groups were tested by the chi-square test using IBM SPSS Statistics 22.0, with two-sided *P*-value <0.05 considered significant.

The pooled K-M curves were plotted and analyzed using the package MetaSurv of software R version 3.6.3 (64). Heterogeneity was assessed by *H* statistic, with *H <*1.2 considered as being indicative of insignificant heterogeneity (65).

The fixed-effects model was used for analysis on the premise of low heterogeneity between studies; otherwise, the random-effects model was applied to pooled results.

## Results

### Study Selection and Characteristics

**Figure 1:**
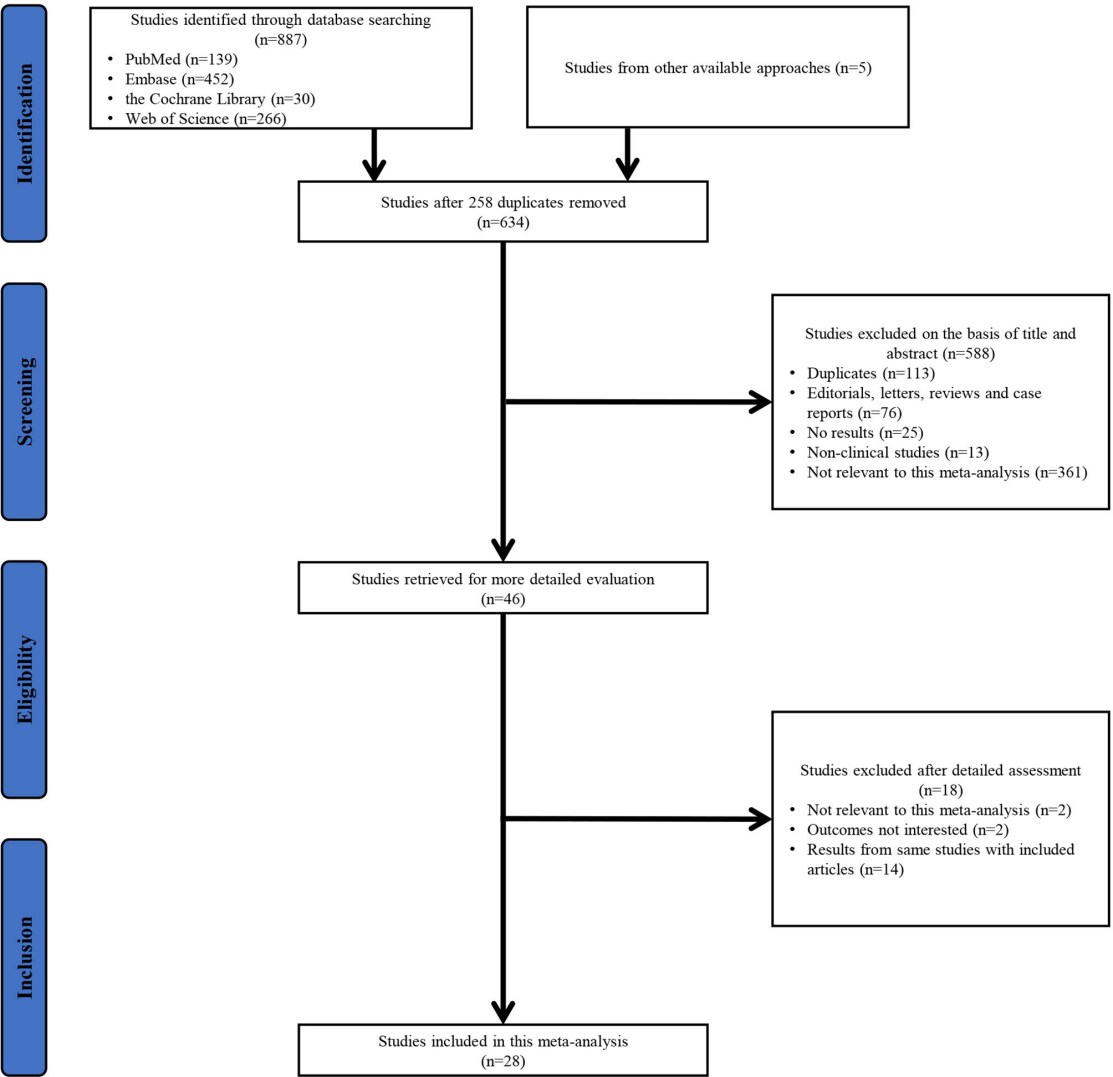
PRISMA flowchart of the study selection process. PRISMA, Preferred Reporting Items for Systematic Reviews and Meta-Analyses.

As shown in **Figure 1**, 887 studies were obtained through database searching and an additional five studies were found in other sources (reference lists of reviews, ASCO meetings, and ESMO meetings). Therefore, a total of 892 studies were identified. After removing duplicates, screening the title and abstract of the remaining studies, and assessing potentially relevant studies in detail, 28 studies were included in this meta-analysis.

Twenty-eight studies involved 1,338 participants and 34 sets of data (five studies had more than one subgroup of interest (39, 43, 47, 48, 60), and we distinguished different subgroups by numbers, such as Oh2020[1], Oh2020[2], and Oh2020[3]). **Tables 1**, **2** provide details of the baseline characteristics and main outcomes of the included studies, respectively.

**Table 1:** Baseline characteristics of included studies with anti-PD1/PDL1 in aBTC.

Study	Region	Study type	Median follow-up, months	Disease status	Drug	Clinical setting	Line of therapy	Sample size	Median age (range), years	Male, %
Kim et al. (32)/NCT02829918	USA	Open-label, multi-institutional, single-group, phase 2	12.4	Advanced refractory BTC	Nivolumab	240 mg, i.v., Q2W for 16 weeks, and then 480 mg, i.v., Q4W	2nd line and beyond	54	65 (28–86)	50
Ueno et al. (39)/JapicCTI-153098	Japan	Open-label, multicenter, non-randomized, phase 1	5.1	Unresectable or recurrent BTC	1) Nivolumab	240 mg, i.v., Q2W	2nd line and beyond	30	68.0	60
			8.2	Unresectable or recurrent BTC	2) Nivolumab + GemCis	Nivolumab 240 mg, i.v., Q2W + cisplatin 25 mg/m^2^, i.v. + gemcitabine 1,000 mg/m^2^, i.v.	1st-line	30	67.5	47
Lee et al. (42)	Korea	Multicenter retrospective study	3.8	PDL1-positive GemCis-refractory BTC	Pembrolizumab	200 mg, i.v., Q3W	2nd line and beyond	51	66 (43–83)	56.9
Kang et al. (33)/NCT03695952	Korea	Single-center, prospective cohort study	9.6	PDL1-positive advanced refractory BTC	Pembrolizumab	200 mg, i.v., Q3W	2nd line and beyond	40	61 (41–76)	57.5
KEYNOTE-028/NCT02054806 (40, 41)	NR	Open-label, multicenter, non-randomized, phase 1b	5.7	aBTC	Pembrolizumab	10 mg/kg, Q2W for ≤2 years	2nd line and beyond	24	64 (43–70)	58.3
KEYNOTE-158/NCT02628067 (40)	NR	Open-label, multicenter, non-randomized, phase 2	7.5	aBTC	Pembrolizumab	200 mg, Q3W	2nd line and beyond	104	63 (34–81)	49.0
Sun et al. (43)	China	Single-center, retrospective study	NR	aBTC	1) PD1 inhibitor monotherapy	NR	2nd line and beyond	20	NR	55
			NR	aBTC	2) PD1 inhibitor + chemotherapy	NR	2nd line and beyond	38	NR	63.2
Yarchoan et al. (46)/NCT03201458	USA	Randomized, open-label, multicenter, phase 2	NR	aBTC	atezolizumab	840 mg, i.v., Q2W	2nd line and beyond	39	NR	NR
Ioka et al. (48)/NCT01938612	Asia	Open-label, multicenter, phase 1	NR	aBTC	1) Durvalumab	10 mg/kg, Q2W	2nd line and beyond	42	64	NR
			NR	aBTC	2) Durvalumab + tremelimumab	durvalumab 20 mg/kg + tremelimumab 1.0 mg/kg, Q4W	2nd line and beyond	65	62	NR
Yoo et al. (31)/NCT02699515	Japan, Korea, Taiwan	Open-label, phase 1	15.3	Metastatic or locally advanced BTC	Bintrafusp alpha	1,200 mg, i.v., Q2W	2nd line and beyond	30	67	63
Merck et al. (49)/NCT03833661	NR	Open-label, multicenter, single-group, phase 2	NR	Locally advanced or metastatic BTC	Bintrafusp alpha	1,200 mg, i.v., Q2W	2nd line	159	NR	NR
Villanueva et al. (51)/NCT03797326	NR	Open-label, non-randomized, phase 2	NR	aBTC	Pembrolizumab + lenvatinib	Pembrolizumab 200 mg, Q3W + lenvatinib 20 mg, q.d.	2nd line and beyond	31	NR	NR
Lin et al. (35)/NCT03895970	NR	Single-arm	9.5	aBTC	Pembrolizumab + lenvatinib	Pembrolizumab 200 mg, Q3W (*n* = 11) or 3 mg/kg, Q3W (*n* = 21) + lenvatinib 12 mg (body weight ≥ 60 kg) or 8 mg (body weight < 60 kg), p.o., q.d.	2nd line and beyond	32	56.5	56
Arkenau et al. (36)/NCT02443324	5 countries	Open-label, multicenter, non-randomized, phase 1	15.7	Previously treated advanced or metastatic BTC	Pembrolizumab + ramucirumab	Pembrolizumab 200 mg, i.v., d1, Q3W + ramucirumab 8 mg/kg, i.v., d1, d8	2nd line and beyond	26	63 (36–78)	30.8
Wang et al. (34)/NCT04642664	China	Open-label, single-center, non-randomized, prospective	13.4	Previously treated aBTC	Camrelizumab + apatinib	Camrelizumab 200 mg, i.v., Q3W + apatinib 250 mg, p.o., q.d.	2nd line and beyond	22	60 (39–72)	52.4
Zong et al. (52)/ChiCTR1900022003	China	Phase 2	8.76	Previously treated aBTC	Sintilimab + anlotinib	Sintilimab 200 mg, i.v., d1, Q3W + anlotinib 12 mg, p.o., d1~d14, Q3W	2nd line	17	59 (43–69)	52.9
Zhou et al. (50)/NCT03996408	China	Open-label, dose-escalating, dose-expansion, phase 1b	NR	Advanced refractory BTC	TQB2450 + anlotinib	Anlotinib 10 mg and then 12 mg, p.o., d1~d14, Q3W + TQB2450 1,200 mg, i.v., d1, Q3W	2nd line and beyond	25	NR	NR
Sun et al. (53)/NCT03825705	China	phase 1b	14.9	aBTC	TQB2450 + anlotinib	Anlotinib 10 mg (*n* = 22) or 12 mg (*n* = 12), d1~d14, Q3W + TQB2450 1,200 mg, Q3W	2nd line	34	57 (37–72)	44.1
Cousin et al. (54)/NCT03475953	France	Open-label, multicenter, single-arm, phase 2	9.8	Advanced refractory BTC	Avelumab + regorafenib	Regorafenib 160 mg, q.d., d1~d21, Q4W + avelumab 10 mg/kg, Q2W	2nd line and beyond	34	63 (36–80)	NR
Oh et al. (47)/NCT03046862	Korea	Phase 2	28.5	Chemo-naive aBTC	1) Durvalumab + tremelimumab + GemCis (biomarker cohort)	Gemcitabine 1,000 mg/m^2^ + cisplatin 25 mg/m^2^, d1, d8, followed by GemCis + durvalumab 1,120 mg + tremelimumab 75 mg, Q3W	1st line	30	NR	NR
			11.9	Chemo-naive aBTC	2) Durvalumab + tremelimumab + GemCis	NR	1st line	46	NR	NR
			11.3	Chemo-naive aBTC	3) Durvalumab + GemCis	NR	1st line	45	NR	NR
Boileve et al. (60)/NCT03704480	France	Safety run-in results of the randomized IMMUNOBIL PRODIGE 57 phase 2 trial	NR	aBTC	1) Durvalumab + tremelimumab	Durvalumab 1,500 mg, i.v., d1 + tremelimumab 75 mg, i.v., d1, Q4W	2nd line	10	67 (60–75)	50
			9.8	aBTC	2) Durvalumab + tremelimumab + paclitaxel	Durvalumab 1,500 mg, i.v., d1 + tremelimumab 75 mg, i.v., d1, Q4W + paclitaxel 80 mg/m^2^, i.v., d1, d8, d15	2nd line	10	70 (61–75)	70
Floudas et al. (55)/NCT02821754	USA	Non-randomized, phase 2	NR	aBTC	Durvalumab + tremelimumab	Tremelimumab 75 mg, Q4W + durvalumab 1,500 mg for 4 doses, followed by durvalumab monotherapy 1,500 mg, Q4W	NR	12	NR	NR
Klein et al. (37)/NCT02923934	Australia	Open-label, multicenter, non-randomized, phase 2	NR	aBTC	Nivolumab + ipilimumab	Nivolumab 3 mg/kg + ipilimumab 1 mg/kg, Q3W for 4 doses, followed by nivolumab monotherapy 3 mg/kg, Q2W	2nd line and beyond	39	65 (37–81)	51
Chiang et al. (58)/NCT04172402	Taiwan	Single arm, phase 2	6.4	aBTC	Nivolumab + GS	Nivolumab 240 mg + gemcitabine 800 mg/m^2^, d1 + S-1 80/100/120 mg, q.d., d1~d10, Q2W	1st line	48	66 (30–80)	46
Liu et al. (56)/NCT03796429	China	Open-label, phase 2	10	aBTC	Toripalimab + GS	Toripalimab 240 mg, i.v., Q3W + gemcitabine 1,000 mg/m^2^, i.v., d1, d8 + S-1 40–60 mg, b.i.d. * 14 days, Q3W	1st line	39	64	48.7
Chen et al. (38)/NCT03486678	China	Open-label, single-arm, phase 2	11.8	aBTC	Camrelizumab + GEMOX	Camrelizumab 3 mg/kg, total dose ≤200 mg, i.v. drip, d1 + gemcitabine 800 mg/m^2^, i.v. drip, d1 + oxaliplatin 85 mg/m^2^, i.v. drip, d2, Q2W	1st line	37	64 (41–74)	70.3
Qin et al. (57)/NCT0309289	China	Multicenter, single-arm, phase 2	NR	aBTC	Camrelizumab + FOLFOX4 or GEMOX	Camrelizumab 3 mg/kg, i.v., Q2W + typical FOLFOX4 or GEMOX	1st line	43	NR	NR
Gou et al. (59)	China	Retrospective study	NR	aBTC	PD1 inhibitors + nab-paclitaxel + S-1	NR	1st line	32	NR	NR

PD1, programmed cell death protein 1; PDL1, programmed cell death ligand 1; aBTC, advanced biliary tract cancer; BTC, biliary tract cancer; USA, United States; GemCis, gemcitabine + cisplatin; S-1, tegafur-gimeracil-oteracil; GS, gemcitabine + tegafur-gimeracil-oteracil; GEMOX, gemcitabine + oxaliplatin; FOLFOX4, fluorouracil + leucovorin + oxaliplatin; i.v., intravenously; i.v. drip, intravenous drips; p.o., orally; q.d., once daily; b.i.d., twice daily; Q2W, every 2 weeks; Q3W, every 3 weeks; Q4W, every 4 weeks; d1, day 1; d2, day 2; d8, day 8; d10, day 10; d14, day 14; d15, day 15; d21, day 21; 1st, first; 2nd, second; NR, not reported.

**Table 2:** Main outcomes extracted from included studies with anti-PD1/PDL1 in aBTC.

Study	Sample size	mPFS (95% CI), months	6m-PFS, %	12m-PFS, %	mOS (95% CI), months	6m-OS, %	12m-OS, %	ORR, %	DCR, %	CR, %	PR, %	SD, %	Any-grade AEs, %	Grade 3–4 AEs, %
Kim et al. (32)/NCT02829918	54	3.68 (2.3–5.69)	NR	NR	14.24 (5.98–NE)	NR	NR	11	50	0	11	39	NR	17
Ueno et al. (39)/JapicCTI-153098	(1) 30	1.4 (1.4–1.4)	NR	NR	5.2 (4.5–8.7)	NR	NR	3	23	0	3	20	57	10
	(2) 30	4.2 (2.8–5.6)	NR	NR	15.4 (11.8–NE)	NR	NR	37	63	0	37	27	100	90
Lee et al. (42)	51	2.1 (1.7–2.4)	NR	NR	6.9 (5.4–8.3)	NR	NR	9.8	35.3	0	9.8	25.5	58.8	7.8
Kang et al. (33)/NCT03695952	40	1.5 (0.0–3.0)	13.1	NR	4.3 (3.5–5.1)	27.5	NR	10.0	47.5	0	10.0	37.5	20.5	0
KEYNOTE-028/NCT02054806 (40, 41)	24	1.8 (1.4–3.1)	13.0	13.0	5.7 (3.1–9.8)	45.8	20.8	13.0	26.1	0	13.0	13.0	66.7	16.7
KEYNOTE-158/NCT02628067 (40)	104	2.0 (1.9–2.1)	11.4	5.2	7.4 (5.5–9.6)	56.4	32.7	5.8	22.1	0	5.8	16.3	54.8	12.5
Sun et al. (43)	(1) 20	2.2 (1.10–3.30)	NR	NR	4.1 (2.79–5.42)	NR	NR	0	65	0	0	65	20.0	5.0
	(2) 38	5.1 (3.59–6.61)	NR	NR	14.9 (10.73–19.07)	NR	NR	34.2	89.5	7.9	26.3	55.3	76.3	34.2
Yarchoan et al. (46)/NCT03201458	39	1.87	NR	NR	NR	NR	NR	2.9	32.4	0	2.9	29.4	NR	NR
Ioka et al. (48)/NCT01938612	(1) 42	NR	NR	NR	8.1 (5.6–10.1)	NR	NR	4.8	16.7	0	4.8	11.9	64	NR
	(2) 65	NR	NR	NR	10.1 (6.2–11.4)	NR	NR	10.8	32.2	0	10.8	21.5	82	NR
Yoo et al. (31)/NCT02699515	30	2.5 (1.3–5.6)	32	24	12.7 (6.7–15.7)	73	52	20	40	7	13	20	63	37
Merck et al. (49)/NCT03833661	159	NR	NR	NR	NR	NR	NR	10.1	NR	NR	NR	NR	NR	NR
Villanueva et al. (51)/NCT03797326	31	6.1 (2.1‒6.4)	NR	NR	8.6 (5.6–NE)	NR	NR	10	68	0	10	58	97	48
Lin et al. (35)/NCT03895970	32	4.9 (4.7–5.2)	33.7	6.25	11.0 (9.6–12.3)	71.9	39.4	25	78.1	0	25	53	100	62.5
Arkenau et al. (36)/NCT02443324	26	1.64 (1.38–2.76)	18.0	NR	6.44 (4.17–13.27)	61.8	30.0	3.8	38.5	0	3.8	34.6	NR	38.5
Wang et al. (34)/NCT04642664	22	4.4 (2.4–6.3)	NR	NR	13.1 (8.1–18.2)	NR	NR	19.0	71.4	0	19.0	52.3	100	63.6
Zong et al. (52)/ChiCTR1900022003	17	6.5 (3.6–9.4)	NR	NR	Not reached	NR	NR	40.0	86.7	NR	NR	46.7	70.6	NR
Zhou et al. (50)/NCT03996408	25	8	NR	NR	NR	NR	NR	41.67	75	12.5	29.2	33.3	83.3	16.7
Sun et al. (53)/NCT03825705	34	5.95 (3.78–11.50)	NR	NR	NR	NR	64.71	11.8	76.5	0	11.8	64.7	NR	NR
Cousin et al. (54)/NCT03475953	34	2.5 (1.9–5.5)	NR	NR	11.9 (6.2–NE)	NR	NR	13.8	51.7	0	13.8	37.9	NR	NR
Oh et al. (47)/NCT03046862	(1) 30	13.0	NR	NR	15.0	NR	NR	50.0	96.7	NR	NR	46.7	NR	NR
	(2) 46	11.9	NR	NR	20.7	NR	NR	73.3	97.8	NR	NR	23.9	NR	NR
	(3) 45	11.0	NR	NR	18.1	NR	NR	73.4	100	NR	NR	26.7	NR	NR
Boileve et al. (60)/NCT03704480	(1) 10	NR	NR	NR	NR	NR	NR	NR	NR	NR	NR	NR	NR	40
	(2) 10	NR	NR	NR	NR	NR	NR	10	60	10	0	50	NR	60
Floudas et al. (55)/NCT02821754	12	3.1 (0.8–4.6)	NR	NR	5.45 (4.60–8.3)	NR	NR	0	41.7	0	0	41.7	NR	NR
Klein et al. (37)/NCT02923934	39	2.9 (2.2–4.6)	NR	NR	5.7 (2.7–11.9)	NR	NR	23	44	0	23	21	NR	NR
Chiang et al. (58)/NCT04172402	48	8.0 (5.8–not reached)	NR	NR	Not reached (10.7–not reached)	NR	NR	41.7	87.5	NR	NR	45.8	NR	NR
Liu et al. (56)/NCT03796429	39	6.7	NR	NR	NR	NR	NR	20.6	85.3	0	20.6	64.7	NR	NR
Chen et al. (38)/NCT03486678	37	6.1 (5.1–6.8)	50	NR	11.8 (8.3–15.4)	NR	NR	54	89	0	54	35	97	70
Qin et al. (57)/NCT03092895	47	NR	NR	NR	NR	NR	NR	7.0	67.4	NR	NR	60.5	NR	NR
Gou et al. (59)	32	5.43	NR	NR	NR	NR	NR	25	84.3	NR	NR	59.4	NR	NR

All patients in most studies were evaluated except that the tumor responses were 46/54 in the Kim2020 study, 23/24 in the KEYNOTE-028 study, 34/39 in the Yarchoan2020 study, 21/22 in the Wang2021 study, 15/17 in the Zong2021 study, 24/25 in the Zhou2021 study, 29/34 in the Cousin2021 study, 34/39 in the Liu2020 study, and 43/47 in the Qin2019 study; PFS was 23/24 in the KEYNOTE-028 study and 21/22 in the Wang2021 study; OS was 21/22 in the Wang2021 study; and safety results were 39/40 in the Kang2020 study and 24/25 in the Zhou2021 study. Five studies had more than one subgroup of interest. Specifically, patients were allocated to the nivolumab group [Ueno20191)] or the nivolumab/GemCis group [Ueno2019(2)] in the Ueno2019 study; the PD1 inhibitor monotherapy group [Sun2019(1)] or the PD1 inhibitor plus chemotherapy group [Sun2019(2)] in the Sun2019 study; the durvalumab group [Ioka2019(1)] or the durvalumab/tremelimumab group [loka2019(2)] in the Ioka2019 study; the biomarker group [receiving durvalumab/tremelimumab with GemCis, [Oh2020(1)], the durvalumab/tremelimumab with GemCis group [Oh2020(2)] or the durvalumab with GemCis group [Oh2020(3)] in the Oh2020 study; the durvalumab/tremelimumab group [Boileve2021(1)] or the durvalumab/tremelimumab with paclitaxel group [Boileve2021(2)] in the Boileve2021 study.

PD1, programmed cell death protein 1; PDL1, programmed cell death ligand 1; aBTC, advanced biliary tract cancer; mPFS, medium progression-free survival; CI, confidence interval; 6m-PFS, 6-month progression-free survival; 12m-PFS, 12-month progression-free survival; mOS, medium overall survival; 6m-OS, 6-month overall survival; 12m-OS, 12-month overall survival; ORR, objective response rate; DCR, disease control rate; CR, complete response; PR, partial response; SD, stable disease; AEs, adverse events; NR, not reported; NE, not estimable; PFS, progression-free survival; OS, overall survival; GemCis, gemcitabine + cisplatin.

### Quality Assessment

Thirteen studies were performed with quality appraisal (**Supplementary Table 1**). Eleven prospective studies [including 1 non-randomized comparative study (39) and 10 single-arm studies (31–38, 40, 41)] evaluated by ROBINS-I were all at moderate risk of bias, thereby meeting the inclusion criteria. Two retrospective studies (42, 43) assessed by the JBI tool were also included in this meta-analysis.

### Efficacy

#### Anti-PD1/PDL1 Monotherapy or in Combination With Other Therapies

**Figure 2:**
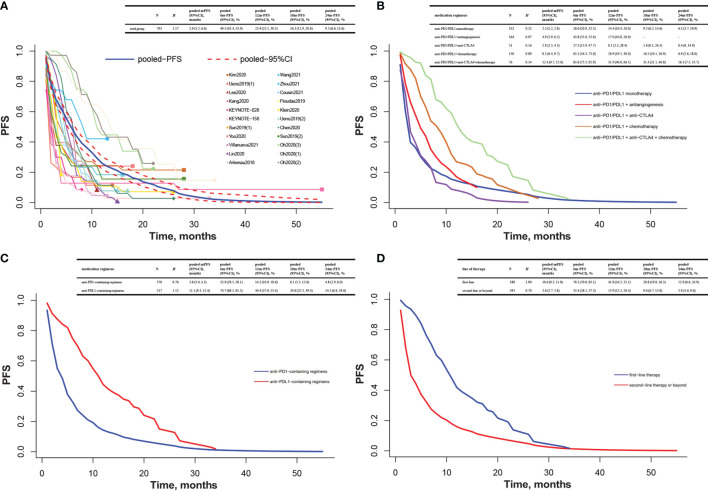
Pooled Kaplan–Meier estimate of PFS. **(A)** Total group; **(B)** anti-PD1/PDL1 monotherapy, anti-PD1/PDL1 combined with antiangiogenesis or anti-CTLA4 or chemotherapy, or combination of anti-PD1/PDL1, anti-CTLA4, and chemotherapy; **(C)** anti-PD1-containing regimens and anti-PDL1-containing regimens; **(D)** first-line therapy and second-line therapy or beyond. Note: Heterogeneity was assessed by *H* statistic, with *H <*1.2 considered as being indicative of insignificant heterogeneity. Three studies had more than one subgroup of interest. Specifically, patients were allocated to the nivolumab group [Ueno2019(1)] or the nivolumab/GemCis group [Ueno2019(2)] in the Ueno2019 study; the PD1 inhibitor monotherapy group [Sun2019(1)] or the PD1 inhibitor plus chemotherapy group [Sun2019(2)] in the Sun2019 study; the biomarker group [receiving durvalumab/tremelimumab with GemCis, [Oh2020(1)], the durvalumab/tremelimumab with GemCis group [Oh2020(2)], or the durvalumab with GemCis group [Oh2020(3)] in the Oh2020 study. PFS, progression-free survival; mPFS, medium progression-free survival; CI, confidence interval; 6m-PFS, 6-month progression-free survival; 12m-PFS, 12-month progression-free survival; 18m-PFS, 18-month progression-free survival; 24m-PFS, 24-month progression-free survival; PD1, programmed cell death protein 1; PDL1, programmed cell death ligand 1; CTLA4, cytotoxic T lymphocyte antigen 4; GemCis, gemcitabine + cisplatin.

**Figure 3:**
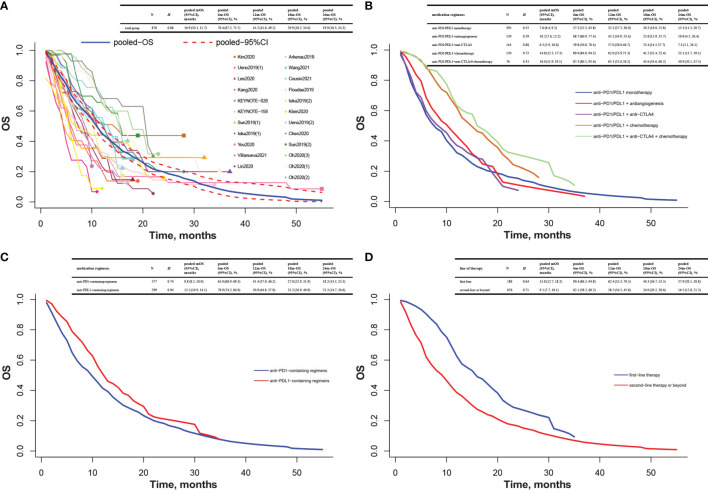
Pooled Kaplan–Meier estimate of OS. **(A)** Total group; **(B)** anti-PD1/PDL1 monotherapy, anti-PD1/PDL1 combined with antiangiogenesis or anti-CTLA4 or chemotherapy, or combination of anti-PD1/PDL1, anti-CTLA4, and chemotherapy; **(C)** anti-PD1-containing regimens and anti-PDL1-containing regimens; **(D)** first-line therapy and second-line therapy or beyond. Heterogeneity was assessed by *H* statistic, with *H <*1.2 considered as being indicative of insignificant heterogeneity. Four studies had more than one subgroup of interest. Specifically, patients were allocated to the nivolumab group [Ueno2019(1)] or the nivolumab/GemCis group [Ueno2019(2)] in the Ueno2019 study; the PD1 inhibitor monotherapy group [Sun2019(1)] or the PD1 inhibitor plus chemotherapy group [Sun2019(2)] in the Sun2019 study; the durvalumab group [Ioka2019(1)] or the durvalumab/tremelimumab group [Ioka2019(2)] in the Ioka2019 study; the biomarker group [receiving durvalumab/tremelimumab with GemCis, Oh2020(1)], the durvalumab/tremelimumab with GemCis group [Oh2020(2)], or the durvalumab with GemCis group [Oh2020(3)] in the Oh2020 study. OS, overall survival; mOS, medium overall survival; CI, confidence interval; 6m-OS, 6-month overall survival; 12m-OS, 12-month overall survival; 18m-OS, 18-month overall survival; 24m-OS, 24-month overall survival; PD1, programmed cell death protein 1; PDL1, programmed cell death ligand 1; CTLA4, cytotoxic T lymphocyte antigen 4; GemCis, gemcitabine + cisplatin.

The pooled K-M curves were built by data extracted from published K-M curves in 19 included studies (31–43, 47, 48, 50, 51, 54, 55). The pooled mPFS was 5.9 months (95% CI 5.2 to 6.6), and the estimated PFS rates were 49.5% at 6 months and 25.9% at 12 months (**Figure 2A**). The pooled mOS was 10.9 months (95% CI 10.1 to 11.7), and the 6- and 12-month OS rates were 70.4% and 45.2%, respectively (**Figure 3A**).

Among patients treated with anti-PD1/PDL1 monotherapy, the pooled mPFS was 2.5 months (95% CI 2.2 to 2.8), and the estimated PFS rates were 26.0% at 6 months and 14.4% at 12 months. The anti-PD1/PDL1 plus anti-CTLA4 group ended up with a similar mPFS of 2.9 months (95% CI 2.3 to 4.5), and the 6- and 12-month PFS rates were 27.5% and 8.1%, respectively. For patients receiving anti-PD1/PDL1 plus antiangiogenesis, the pooled mPFS was 4.9 months (95% CI 3.9 to 6.2), and the estimated PFS rates were 43.8% at 6 months and 17.0% at 12 months. For patients taking anti-PD1/PDL1 plus chemotherapy, the pooled mPFS was 8.2 months (95% CI 6.4 to 9.7), and the estimated PFS rates were 63.1% at 6 months and 26.9% at 12 months. Anti-PD1/PDL1 combined with anti-CTLA4 and chemotherapy elicited a longer mPFS of 12.4 months (95% CI 9.7 to 15.0), and the 6- and 12-month PFS rates were 83.8% and 51.9%, respectively (**Figure 2B**).

A similar trend was found in mOS. Anti-PD1/PDL1 monotherapy and anti-PD1/PDL1 plus anti-CTLA4 were very much similar [7.6 months (95% CI 6.4 to 9.2) vs. 8.3 months (95% CI 5.9 to 10.8)]. Anti-PD1/PDL1 plus antiangiogenesis reported a longer mOS of 10.2 months (95% CI 7.6 to 12.2), while the most impressive mOS was observed in anti-PD1/PDL1 plus chemotherapy with or without anti-CTLA4 [anti-PD1/PDL1 + chemotherapy + anti-CTLA4: 16.0 months (95% CI 11.9 to 19.1); anti-PD1/PDL1 + chemotherapy: 14.8 months (95% CI 12.3 to 17.5)]. The 6- and 12-month OS rates were 57.2% and 32.2% in anti-PD1/PDL1 monotherapy, 59.8% and 37.0% in anti-PD1/PDL1 plus anti-CTLA4, 68.7% and 43.2% in anti-PD1/PDL1 plus antiangiogenesis, 89.0% and 62.0% in anti-PD1/PDL1 plus chemotherapy, and 87.5% and 63.3% in anti-PD1/PDL1 combined with anti-CTLA4 and chemotherapy, respectively (**Figure 3B**).

**Figure 4:**
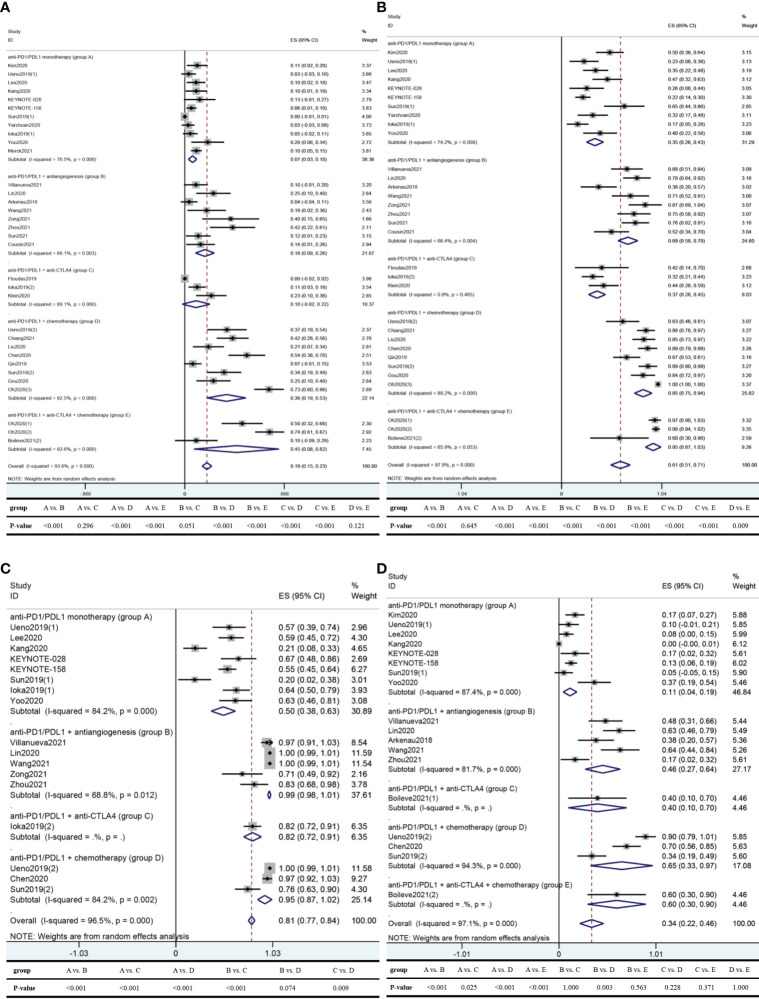
Pooled results of ORR, DCR, any-grade AEs, and grade 3–4 AEs in total and by medication regimen subgroup. **(A)** ORR; **(B)** DCR; **(C)** any-grade AEs; **(D)** grade 3–4 AEs. Five studies had more than one subgroup of interest. Specifically, patients were allocated to the nivolumab group [Ueno2019(1)] or the nivolumab/GemCis group [Ueno2019(2)] in the Ueno2019 study; the PD1 inhibitor monotherapy group [Sun2019(1)] or the PD1 inhibitor plus chemotherapy group [Sun2019(2)] in the Sun2019 study; the durvalumab group [Ioka2019(1)] or the durvalumab/tremelimumab group [Ioka2019(2)] in the Ioka2019 study; the biomarker group [receiving durvalumab/tremelimumab with GemCis, Oh2020(1)], the durvalumab/tremelimumab with GemCis group [Oh2020(2)], or the durvalumab with GemCis group [Oh2020(3)] in the Oh2020 study; the durvalumab/tremelimumab group [Boileve2021(1)] or the durvalumab/tremelimumab with paclitaxel group [Boileve2021(2)] in the Boileve2021 study. Note: Heterogeneity across studies was evaluated by the Cochran *Q* chi-square test and *I* ^2^ statistic, with *P <*0.1 for the *Q* test deemed to have high heterogeneity and *I* ^2^ >50% regarded as an indicator of moderate-to-high heterogeneity. If separate verdicts from the *Q* test and *I* ^2^ statistic were at opposite poles, we would give priority to the conclusion from the *I* ^2^ statistic since the former is proverbially underpowered to detect heterogeneity. Differences between groups were tested by the chi-square test using IBM SPSS Statistics 22.0, with two-sided *P*-value <0.05 considered significant. ORR, objective response rate; DCR, disease control rate; AEs, adverse events; ES, effect size; CI, confidence interval; PD1, programmed cell death protein 1; PDL1, programmed cell death ligand 1; CTLA4, cytotoxic T lymphocyte antigen 4; GemCis, gemcitabine + cisplatin.

In 28 studies reporting ORR (31–43, 46–60), the pooled ORR was 19.3%. The pooled ORRs of anti-PD1/PDL1 monotherapy, anti-PD1/PDL1 plus antiangiogenesis, anti-PD1/PDL1 plus anti-CTLA4, anti-PD1/PDL1 plus chemotherapy, and anti-PD1/PDL1 combined with anti-CTLA4 and chemotherapy were 6.8%, 17.5%, 9.9%, 36.3%, and 45.1% respectively, (**Figure 4A**).

There were 27 studies reporting DCR (31–43, 46–48, 50–60), resulting in a pooled DCR of 61.1%. The pooled DCRs of anti-PD1/PDL1 monotherapy, anti-PD1/PDL1 plus antiangiogenesis, anti-PD1/PDL1 plus anti-CTLA4, anti-PD1/PDL1 plus chemotherapy, and anti-PD1/PDL1 combined with anti-CTLA4 and chemotherapy were 34.7%, 68.7%, 36.8%, 84.6%, and 95.0%, respectively (**Figure 4B**).

CR and PR were reported in 22 studies (31–43, 46, 48, 50, 51, 53–56, 60), while SD was reported in 27 studies (31–43, 46–48, 50–60). The pooled CR, PR, and SD in total and by medication regimen subgroup are recorded in **Table 3**.

**Table 3:** Pooled results of CR, PR, SD in total and by medication regimens subgroup with anti-PD1/PDL1 in aBTC.

Medication regimens	CR				PR				SD			
	*N*	ES (95% CI), %	*I* ^2^, %	*P*	*N*	ES (95% CI), %	*I* ^2^, %	*P*	*N*	ES (95% CI), %	*I* ^2^, %	*P*
Anti-PD1/PDL1 monotherapy	420	0.0 (−0.1, 0.2)	0.0	0.989	420	5.9 (2.5, 9.2)	69.8	<0.001	420	26.1 (18.1, 34.1)	74.6	<0.001
Anti-PD1/PDL1 + antiangiogenesis	197	0.0 (−0.2, 0.3)	0.0	0.756	197	13.8 (7.3, 20.3)	49.5	0.065	212	48.0 (39.5, 56.5)	39.5	0.116
Anti-PD1/PDL1 + anti-CTLA4	116	0.0 (−0.2, 0.3)	0.0	0.996	116	9.9 (−2.2, 21.9)	89.1	<0.001	116	22.7 (15.1, 30.2)	0.0	0.379
Anti-PD1/PDL1 + chemotherapy	139	0.0 (−0.3, 0.4)	7.1	0.358	139	33.9 (19.5, 48.3)	72.3	0.013	307	46.5 (35.6, 57.4)	75.8	<0.001
Anti-PD1/PDL1 + anti-CTLA4 + chemotherapy	10	10.0 (−8.6, 28.6)	–	–	10	0.1 (−1.9, 2.1)	–	–	86	37.5 (19.3, 55.7)	63.6	0.064
Overall	882	0.0 (−0.1, 0.1)	0.0	0.995	882	10.1 (7.3, 12.9)	85.1	<0.001	1,141	37.6 (31.6, 43.6)	80.9	<0.001

Heterogeneity across studies was evaluated by the Cochran Q chi-square test and I^2^ statistic, with P <0.1 for the Q test deemed to have high heterogeneity and I^2^ >50% regarded as an indicator of moderate-to-high heterogeneity. If separate verdicts from the Q test and I^2^ statistic were at opposite poles, we would give priority to the conclusion from the I^2^ statistic since the former is proverbially underpowered to detect heterogeneity.

CR, complete response; PR, partial response; SD, stable disease; PD1, programmed cell death protein 1; PDL1, programmed cell death ligand 1; aBTC, advanced biliary tract cancer; ES, effect size; CI, confidence interval; CTLA4, cytotoxic T lymphocyte antigen.

#### Anti-PD1-Containing Regimens and Anti-PDL1-Containing Regimens

Among patients taking anti-PD1-containing regimens, the pooled mPFS was 3.8 months (95% CI 3.0 to 4.3) and the 6- and 12-month PFS rates were 32.9% and 14.2%, respectively. The anti-PDL1-containing regimen group demonstrated a much longer mPFS of 11.1 months (95% CI 9.3 to 12.4), and the 6- and 12-month PFS rates were 74.7% and 44.4%, respectively (**Figure 2C**). Furthermore, the gap in mOS between these two regimens was nearly 3 months [9.8 months (95% CI 8.5 to 10.9) vs. 12.2 months (95% CI 10.9 to 14.1)]. The 6- and 12-month OS rates were 65.0% and 41.4% in the anti-PD1-containing regimen group and 78.9% and 50.9% in the anti-PDL1-containing regimen group, respectively (**Figure 3C**).

Eighteen studies (32–43, 51, 52, 56–59) reported the tumor response of anti-PD1-containing regimens, and 10 studies (31, 46–50, 53–55, 60) described that of anti-PDL1-containing regimens. Anti-PDL1-containing regimens yielded a higher ORR than anti-PD1-containing regimens (23.7% vs. 17.4%, *P*-value = 0.005), albeit an unconspicuous disadvantage in DCR (61.1% vs. 61.3%, *P*-value = 0.933). When combined with antiangiogenesis, anti-PDL1 was also superior to anti-PD1 in ORR (20.3% vs. 16.3%, *P*-value = 0.381), but they were equally matched in DCR (68.4% vs. 68.9%, *P*-value = 0.980). On the contrary, despite a narrow victory in ORR (7.6% vs. 6.2%, *P*-value = 0.474), anti-PDL1 monotherapy was defeated by anti-PD1 monotherapy in DCR (28.4% vs. 37.4%, *P*-value = 0.094). The detailed discrepancies between anti-PD1-containing regimens and anti-PDL1-containing regimens are elaborated in **Table 4**.

**Table 4:** Pooled results of ORR, DCR, any-grade AEs, and grade 3–4 AEs of anti-PD1-containing regimens or anti-PDL1-containing regimens in aBTC.

Medication regimens	ORR					DCR				
	*N*	ES (95% CI), %	*I* ^2^, %	*P*	*P*-value	*N*	ES (95% CI), %	*I* ^2^, %	*P*	*P*-value
Overall					0.005					0.933
Anti-PD1	740	17.4 (11.9, 22.8)	89.8	<0.001		740	61.3 (49.4, 73.1)	93.6	<0.001	
Anti-PDL1	560	23.7 (13.8, 33.6)	96.1	<0.001		401	61.1 (46.6, 75.5)	97.9	<0.001	
Monotherapy					0.474					0.094
Anti-PD1	314	6.2 (1.8, 10.5)	74.9	0.001		314	37.4 (26.1, 48.7)	77.9	<0.001	
Anti-PDL1	265	7.6 (2.3, 12.8)	58.5	0.065		106	28.4 (14.1, 42.8)	65.0	0.058	
Combined with antiangiogenesis					0.381					0.980
Anti-PD1	125	16.3 (5.5, 27.0)	69.6	0.011		125	68.9 (53.7, 84.1)	74.8	0.003	
anti-PDL1	87	20.3 (5.2, 35.5)	72.1	0.028		87	68.4 (53.3, 83.4)	59.8	0.083	
Combined with anti-CTLA4					0.010					0.301
Anti-PD1	39	23.1 (9.9, 36.3)	–	–		39	43.6 (28.0, 59.2)	–	–	
Anti-PDL1	77	4.8 (-5.6, 15.2)	86.4	0.007		77	33.6 (23.1, 44.2)	0.0	0.543	
Combined with chemotherapy					<0.001					0.002
Anti-PD1	262	30.7 (17.2, 44.2)	86.0	<0.001		262	82.6 (75.9, 89.3)	56.2	0.033	
Anti-PDL1	45	73.3 (60.4, 86.3)	–	–		45	100.0 (99.5, 100.4)	–	–	
Combined with anti-CTLA4 + chemotherapy					–					–
Anti-PD1	–	–	–	–		–	–	–	–	
Anti-PDL1	86	45.1 (8.1, 82.1)	93.6	<0.001		86	95.0 (87.0, 103.0)	65.9	0.053	
**Medication regimens**	**Any-grade AEs**					**Grade 3–4 AEs**				
	***N***	**ES (95% CI), %**	***I* ^2^, %**	***P***	***P*-value**	***N***	**ES (95% CI), %**	***I* ^2^, %**	***P***	***P*-value**
Overall					0.017					0.750
Anti-PD1	475	82.5 (78.8, 86.3)	97.0	<0.001		538	33.3 (19.4, 47.2)	97.6	<0.001	
Anti-PDL1	161	74.2 (63.9, 84.5)	56.1	0.077		74	35.1 (17.6, 52.7)	61.5	0.050	
Monotherapy					0.008					<0.001
Anti-PD1	268	46.1 (30.3, 61.9)	86.4	<0.001		322	9.0 (2.6, 15.4)	84.4	<0.001	
Anti-PDL1	72	63.9 (52.8, 75.0)	0.0	0.934		30	36.7 (19.4, 53.9)	–	–	
Combined with antiangiogenesis					0.001					0.001
Anti-PD1	102	99.7 (98.4, 101.0)	62.8	0.045		111	53.2 (41.6, 64.9)	38.6	0.180	
Anti-PDL1	24	83.3 (68.4, 98.2)	–	–		24	16.7 (1.8, 31.6)	–	–	
Combined with anti-CTLA4					–					–
Anti-PD1	–	–	–	–		–	–	–	–	
Anti-PDL1	65	81.5 (72.1, 91.0)	–	–		10	40.0 (9.6, 70.4)	–	–	
Combined with chemotherapy					–					–
Anti-PD1	105	94.6 (87.0, 102.2)	84.2	0.002		105	65.1 (32.8, 97.5)	94.3	<0.001	
Anti-PDL1	–	–	–	–		–	–	–	–	
Combined with anti-CTLA4 + chemotherapy					–					–
Anti-PD1	–	–	–	–		–	–	–	–	
Anti-PDL1	–	–	–	–		10	60.0 (29.6, 90.4)	–	–	

Heterogeneity across studies was evaluated by the Cochran Q chi-square test and I^2^ statistic, with P <0.1 for the Q test deemed to have high heterogeneity and I^2^ >50% regarded as an indicator of moderate-to-high heterogeneity. If separate verdicts from the Q test and I^2^ statistic were at opposite poles, we would give priority to the conclusion from the I^2^ statistic since the former is proverbially underpowered to detect heterogeneity. Differences between groups were tested by the chi-square test using IBM SPSS Statistics 22.0, with two-sided P-value <0.05 considered significant.

ORR, objective response rate; DCR, disease control rate; AEs, adverse events; PD1, programmed cell death protein 1; PDL1, programmed cell death ligand 1; aBTC, advanced biliary tract cancer; ES, effect size; CI, confidence interval; CTLA4, cytotoxic T lymphocyte antigen 4.

#### First-Line Therapy and Second-Line Therapy or Beyond

The mPFS was 10.6 months (95% CI 9.2 to 11.8) for first-line therapy and 3.0 months (95% CI 2.7 to 3.8) for second-line therapy or beyond. The 6- and 12-month PFS rates were 76.3% and 41.8% for first line and 32.4% and 15.9% for second line or beyond (**Figure 2D**). Likewise, the mOS was also longer at first-line settings than at second-line settings or beyond [15.8 months (95% CI 12.7 to 18.2) vs. 9.1 months (95% CI 7.7 to 10.1)]. The 6- and 12-month OS rates were 90.4% and 62.4% for first line and 62.1% and 38.5% for second line or beyond (**Figure 3D**).

A comparison of the tumor response between first line and second line or beyond is shown in **Table 5**. At first-line setting, the ORR was obviously higher than that at second-line setting or beyond (42.3% vs. 11.6%, *P*-value < 0.001). The difference in DCR was also significant, with a higher rate being observed in first-line therapy than in second-line therapy or beyond (88.6% vs. 51.1%, *P*-value < 0.001).

**Table 5:** Pooled results of ORR, DCR, any-grade AEs, and grade 3–4 AEs by line of therapy subgroup in aBTC.

Line of therapy subgroup	ORR				DCR				Any-grade AEs				Grade 3–4 AEs			
	*N*	ES (95% CI), %	*I* ^2^, %	*P*	*N*	ES (95% CI), %	*I* ^2^, %	*P*	*N*	ES (95% CI), %	*I* ^2^, %	*P*	*N*	ES (95% CI), %	*I* ^2^, %	*P*
First-line therapy	345	42.3 (24.0, 60.6)	94.2	<0.001	345	88.6 (82.6, 94.5)	87.2	<0.001	67	99.9 (99.3, 100.6)	0.0	0.320	67	80.8 (61.5, 100.1)	77.8	0.034
Second-line therapy or beyond	943	11.6 (7.9, 15.2)	82.9	<0.001	784	51.1 (40.4, 61.8)	91.5	<0.001	569	72.2 (67.0, 77.3)	97.0	<0.001	545	26.8 (17.7, 35.9)	93.6	<0.001
*P*-value		<0.001				<0.001				<0.001				<0.001		

Heterogeneity across studies was evaluated by the Cochran Q chi-square test and I^2^ statistic, with P <0.1 for the Q test deemed to have high heterogeneity and I^2^ >50% regarded as an indicator of moderate-to-high heterogeneity. If separate verdicts from the Q test and I^2^ statistic were at opposite poles, we would give priority to the conclusion from the I^2^ statistic since the former is proverbially underpowered to detect heterogeneity. Differences between groups were tested by the chi-square test using IBM SPSS Statistics 22.0, with two-sided P-value <0.05 considered significant.

ORR, objective response rate; DCR, disease control rate; AEs, adverse events; aBTC, advanced biliary tract cancer; ES, effect size; CI, confidence interval.

### Safety

There were 14 studies (31, 33–35, 38–43, 48, 50–52) reporting any-grade AEs and 15 studies (31–36, 38–43, 50, 51, 60) reporting grade 3–4 AEs. The overall pooled any-grade AE rate and grade 3–4 AE rate were 80.6% and 34.0% (**Figures 4C, D**). The most frequent any-grade AE was reactive cutaneous capillary endothelial proliferation (RCCEP, 45.1%), followed by hypertension (39.9%), hypoalbuminemia (36.0%), leukopenia (34.0%), decreased appetite (26.2%), and asthenia (25.8%) (**Supplementary Table 2**). Of note, RCCEP and hypoalbuminemia were only reported in two studies using camrelizumab (34, 38). Grade 3–4 AEs that occurred in more than 3% of the patients were hypertension (15.4%), γ-glutamyltransferase increase (9.4%), gastrointestinal hemorrhage (9.3%), elevated bilirubin (8.9%), leukopenia (6.2%), and thrombocytopenia (3.5%) (**Supplementary Table 2**).

Any-grade AEs occurred in 50.5% of the patients in the anti-PD1/PDL1 alone group, 99.4% of the patients taking anti-PD1/PDL1 plus antiangiogenesis, and 94.6% of the patients receiving anti-PD1/PDL1 plus chemotherapy (**Figure 4C**). After omitting two studies using anlotinib (50, 52), the incidence of any-grade AEs in the anti-PD1/PDL1 plus antiangiogenesis group was 99.9% (95% CI 99.4% to 100.4%; *I* ^2^ = 0.0%; *P* = 0.605).

Grade 3–4 AEs occurred in 11.5% of the patients treated with anti-PD1/PDL1 monotherapy, 45.5% of the patients taking anti-PD1/PDL1 plus antiangiogenesis, and 65.1% of the patients receiving anti-PD1/PDL1 plus chemotherapy (**Figure 4D**). After omitting one study using anlotinib (50), the incidence of grade 3–4 AEs in the anti-PD1/PDL1 combined with antiangiogenesis group was 53.2% (95% CI 41.6% to 64.9%; *I* ^2^ = 38.6%; *P* = 0.180).

At the safety evaluation, there were some differences between anti-PD1-containing regimens and anti-PDL1-containing regimens regarding any-grade AE rate (82.5% vs. 74.2%, *P*-value = 0.017) and grade 3–4 AE rate (33.3% vs. 35.1%, *P*-value = 0.750). Patients taking anti-PDL1 monotherapy were much more likely to have any-grade AEs than patients in the anti-PD1 monotherapy group (63.9% vs. 46.1%, *P*-value = 0.008) (**Table 4**). Heterogeneity for any-grade AEs of the anti-PD1 monotherapy group changed significantly after omitting two single-center studies (33, 43), which resulted in 57.5% (95% CI 50.9% to 64.2%; *I* ^2^ = 0.0%; *P* = 0.739). In addition, when Sun2019(1) (43) was removed, there was no evidence of heterogeneity for grade 3–4 AEs of the anti-PD1 monotherapy group [11.8% (95% CI 7.9% to 15.7%; *I* ^2 =^ 0.0%; *P* = 0.629)].

We also saw a significant difference between first-line therapy and second-line therapy or beyond in the incidence of any-grade AEs (99.9% vs. 72.2%, *P*-value < 0.001). Additionally, first-line therapy revealed a higher grade 3–4 AE rate than second-line therapy or beyond (80.8% vs. 26.8%, *P*-value < 0.001) (**Table 5**).

### Sensitivity Analyses

The sensitivity analyses of pooled estimates of tumor response and safety proved to be robust except any-grade AEs in the total group, in the anti-PD1/PDL1 plus antiangiogenesis group, in the anti-PD1 plus antiangiogenesis group, and in the second-line therapy or beyond. **Supplementary Table 3** offers the pooled results after omitting the study that influenced the robustness of pooled any-grade AEs discussed above.

### Publication Bias

Funnel plots and Egger’s tests were conducted in tumor response of the total group, ORR of anti-PD1-containing regimens, and ORR and DCR of second-line therapy or beyond (**Supplementary Figure 1**), all of which included no fewer than 20 sets of data. Except for DCR of second-line therapy or beyond (*P* = 0.235), the results of Egger’s test represented a possibility of publication bias (*P* < 0.001).

## Discussion

To our knowledge, this meta-analysis was the first quantitative analysis to evaluate the efficacy and safety of anti-PD1/PDL1 in aBTC. With a majority of studies being non-comparative, we selectively extracted and analyzed data on survival, tumor response, and safety of the anti-PD1/PDL1 included arm.

On the whole, the pooled mPFS, mOS, ORR, DCR, any-grade AEs, and grade 3–4 AEs of aBTC patients receiving anti-PD1/PDL1 were 5.9 months, 10.9 months, 19.3%, 61.1%, 80.6%, and 34.0%, respectively. Nevertheless, heterogeneity caused by medication regimens and line of therapy came to light through subgroup analyses.

Our results showed that combination regimens with anti-PD1/PDL1 conferred an advantage in PFS, OS, ORR, DCR, PR, and SD over anti-PD1/PDL1 monotherapy. Furthermore, anti-PD1/PDL1 combined with chemotherapy with or without anti-CTLA4 was associated with impressively longer mPFS and mOS than other combination regimens. Likewise, tumor response was also better in this treatment plan. We noticed that the combinations of anti-PD1/PDL1 and chemotherapeutic drugs with or without anti-CTLA4 were mostly used as first-line therapy, while anti-PD1/PDL1 monotherapy or combined with antiangiogenesis or anti-CTLA4 was entirely applied in second-line setting or beyond. Hence, this obvious distinction in efficacy could be partly attributable to the difference in the line of therapy. The compelling safety benefits of anti-PD1/PDL1 monotherapy over combination therapy were observed (*P*-value < 0.05). Despite a slight decrease in the incidence of any-grade AEs compared with anti-PD1/PDL1 plus antiangiogenesis (94.6% vs. 99.4%, *P*-value = 0.074), anti-PD1/PDL1 plus chemotherapy was more likely to develop grade 3–4 AEs (65.1% vs. 45.5%, *P*-value = 0.003). The heterogeneity for any-grade AEs and grade 3–4 AEs of anti-PD1/PDL1 plus antiangiogenesis may result from the use of anlotinib in the Zhou2021 and Zong2021 studies (50, 52), probably because anlotinib is a multitarget tyrosine kinase inhibitor which is known for its more manageable toxicity (66). A phase 3 study of anlotinib plus TQB2450 versus chemotherapy as second-line treatment for aBTC is currently ongoing (NCT04809142).

Overall, anti-PDL1-containing regimens did better than anti-PD1-containing regimens in mPFS (11.1 vs. 3.8 months), mOS (12.2 vs. 9.8 months), ORR (23.7% vs. 17.4%, *P*-value = 0.005), and any-grade AEs (74.2% vs. 82.5%, *P*-value = 0.017), while no significant differences in DCR and grade 3–4 AEs were noted (*P*-value = 0.933 for DCR and *P*-value = 0.750 for grade 3–4 AEs). When used as monotherapy or combined with antiangiogenesis, the differences in ORR and DCR were also insignificant. Intriguingly, the comparison of any-grade AEs between single-agent anti-PD1 and anti-PDL1 was in stark contrast to the overall situation, with a trend favoring anti-PD1 monotherapy (46.1% vs. 63.9%, *P*-value = 0.008). We next sought to probe into the reasons for this phenomenon. Firstly, a much higher any-grade AE rate (>94%) exists in anti-PD1/PDL1 plus antiangiogenesis or chemotherapy (**Figure 4C**). Secondly, from the forest plot of any-grade AEs (**Supplementary Figure 2**), the weight of these two combinations in anti-PDL1-containing regimens was far smaller than that in anti-PD1-containing regimens (23.27% vs. 72.38%). Hence, we fostered the suspicion that the difference in weight gave anti-PDL1-containing regimens an advantage in any-grade AEs over anti-PD1-containing regimens. Regrettably, due to insufficiency of studies, it is utterly premature to jump to the conclusion that anti-PDL1 has an advantage over anti-PD1 in safety when combined with other therapies. For the same reason, the comparisons of tumor response between anti-PD1 and anti-PDL1, when combined with anti-CTLA4 or chemotherapy with or without anti-CTLA4, are getting nowhere. Six studies (33, 39–43) reporting any-grade AEs and seven studies (32, 33, 39–43) reporting grade 3–4 AEs of anti-PD1 monotherapy demonstrated significant heterogeneity, which may be due to the difference between the single-center design of Kang2020 and Sun2019(1) (33, 43) and the multicenter design of the remaining studies. There are several ongoing multicenter phase 3 trials regarding the combination of anti-PD1/PDL1 and chemotherapy in aBTC, such as pembrolizumab plus GemCis (NCT04924062; NCT04003636), durvalumab plus GemCis (NCT03875235), and KN035 plus gemcitabine/oxaliplatin (NCT03478488). We are looking forward to these results.

Regardless of medication regimens, the pooled mPFS, mOS, ORR, DCR, and grade 3–4 AE rate of first-line anti-PD1/PDL1 were 10.6 months, 15.8 months, 42.3%, 88.6%, and 80.8%, which seemed to exhibit superior tumor growth suppression but a greater risk compared with the GemCis group (mPFS: 8.0 months; mOS: 11.7 months; ORR: 26.1%; DCR: 81.4%; grade 3–4 AE rate: 70.7%) (6). The combined toxicity of anti-PD1/PDL1 and chemotherapy took the responsibility for the greater occurrence of AEs in the immunotherapy group. Compared with mFOLFOX (7), anti-PD1/PDL1 serving as second-line therapy or beyond offered potentially preferable efficacy and more satisfactory safety (mOS: 6.2 vs. 9.1 months; ORR: 5% vs. 11.6%; DCR: 33% vs. 51.1%; any-grade AE rate: 99% vs. 72.2%; grade 3–4 AE rate: 60% vs. 26.8%). The unnaturally greater risk of first-line anti-PD1/PDL1 than second-line therapy or beyond might be attributed to its zero weight of the monotherapy group which enjoyed lower incidence of AEs.

So far, there has been no large-scale, phase 3, randomized controlled trial verifying the benefits and risks of the abovementioned regimens in aBTC. Moreover, biomarkers capable of predicting the response to anti-PD1/PDL1 remain understudied, making the identification of reliable biomarkers a pressing task (17). The low incidence of BTC goes against the initiation of clinical trials of large scale, so we recommend multicenter collaborative efforts to bridge the major knowledge gaps. In this meta-analysis, we strictly followed the PRISMA guidelines, made the most efficient use of the available clinical studies, and conducted subgroup analyses as much as possible. We believe this article will generate more powerful evidence on when and how to prescribe anti-PD1/PDL1 for patients with aBTC.

Admittedly, our study still had some limitations. First of all was high heterogeneity which may be caused by methodological and clinical diversities between studies. On the one hand, the design of the included studies differed in several ways, such as the number of centers involved, clinical phase, duration of follow-up, and sample size. Additionally, with most of the studies being single arm, the comparison was based on data from the population with a different baseline, so comparability between studies was somewhat limited. On the other hand, baseline characteristics of participants differed greatly in that BTC is a heterogeneous group of malignancies. Considerable differences of epidemiology, biology, and management exist among the anatomical subtypes (4). Moreover, there were varied clinical interventions such as diverse medication regimens and different lines of therapy, which is why we performed subgroup analyses. The second one was publication bias primarily coming from the overwhelming preferences of sponsors, periodicals, and researchers for positive results. What is more, the significant between-study heterogeneity was another contributing factor to publication bias (63).

## Conclusions

The head-to-head comparative trials concerning anti-PD1/PDL1 in BTC are consistently scarce in the context of increasing incidence of this tumor. Hence, it was timely and necessary to conduct this meta-analysis. Although further studies with control groups are warranted to confirm the efficacy and safety of anti-PD1/PDL1, our findings unequivocally lend support to the use of this treatment in patients with aBTC.

## Data Availability Statement

The original contributions presented in the study are included in the article/**Supplementary Material**. Further inquiries can be directed to the corresponding authors.

## Author Contributions

GG, QJ, and JH designed the study. QJ and XL screened the studies and extracted the data. Quality of evidence was judged by JH and BZ. XC and BC analyzed and interpreted the data. QJ and JH prepared the figures and drafted the manuscript. SL and GG contributed to the review and editing. All authors approved the final version of the article, including the authorship list.

## Funding

This study was funded by the Guangdong Provincial Natural Science Foundation (grant no. 2021A1515012368).

## Conflict of Interest

The authors declare that the research was conducted in the absence of any commercial or financial relationships that could be construed as a potential conflict of interest.

## Publisher’s Note

All claims expressed in this article are solely those of the authors and do not necessarily represent those of their affiliated organizations, or those of the publisher, the editors and the reviewers. Any product that may be evaluated in this article, or claim that may be made by its manufacturer, is not guaranteed or endorsed by the publisher.

## Supplementary Material

Supplementary Figure 1 Funnel plots depicting the publication bias in included studies. **(A)** ORR of total group; **(B)** DCR of total group; **(C)** CR of total group; **(D)** PR of total group; **(E)** SD of total group; **(F)** ORR of anti-PD1-containing regimens; **(G)** ORR of second line therapy or beyond; **(H)** DCR of second line therapy or beyond. ORR, objective response rate; DCR, disease control rate; CR, complete response; PR, partial response; SD, stable disease; PD1, programmed cell death protein 1.

Supplementary Figure 2 Forest plot of any-grade AEs in anti-PD1-containing regimens and anti-PDL1-containing regimens. **(A)** Anti-PD1-containing regimens; **(B)** anti-PDL1-containing regimens. Three studies had more than one subgroup of interest. Specifically, patients were allocated to nivolumab group [Ueno2019(1)] or nivolumab/GemCis group [Ueno2019(2)] in Ueno2019 study; PD1 inhibitor monotherapy group [Sun2019(1)] or PD1 inhibitor plus chemotherapy group [Sun2019(2)] in Sun2019 study; durvalumab group [Ioka2019(1)] or durvalumab/tremelimumab group [Ioka2019(2)] in Ioka2019 study. Heterogeneity across studies was evaluated by the Cochran *Q* chi-square test and *I^2^* statistic, with p<0.1 for the *Q* test deemed to have high heterogeneity and *I^2^* >50% regarded as an indicator of moderate-to-high heterogeneity. If separate verdicts from the *Q* test and *I^2^* statistic were at opposite poles, we would give priority to the conclusion from *I^2^* statistic since the former is proverbially underpowered to detect heterogeneity. AEs, adverse events; ES, effect size; CI, confidence interval; PD1, programmed cell death protein 1; PDL1, programmed cell death ligand 1; CTLA4, cytotoxic T lymphocyte antigen 4; GemCis, gemcitabine + cisplatin.
