# Supplementary figures and images for: Efficacy and Safety of Anti-PD1/PDL1 in Advanced Biliary Tract Cancer: A Systematic Review and Meta-Analysis
Source: Front Immunol. 2022 Mar 2;13:801909. doi: 10.3389/fimmu.2022.801909 (PMC8924050; doi:10.3389/fimmu.2022.801909)

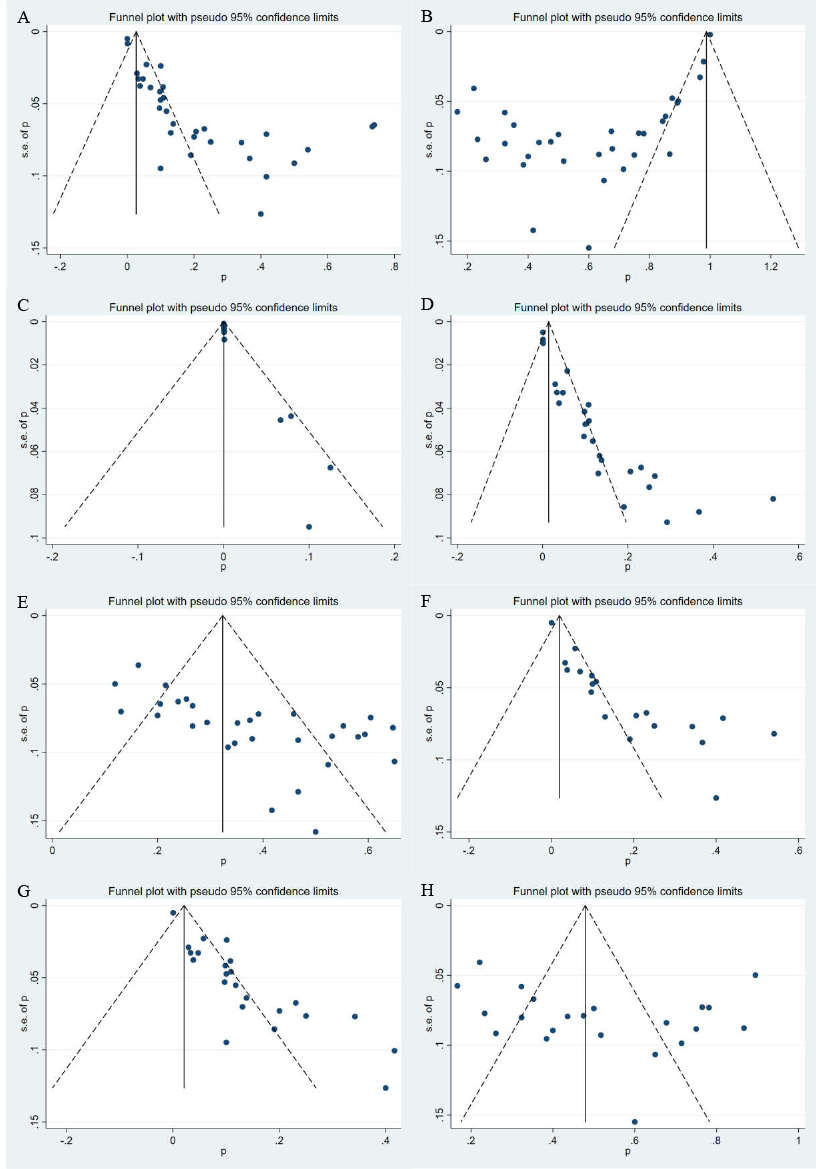

Supplement: Supplementary Figure 1 — Funnel plots depicting the publication bias in included studies. (A) ORR of total group; (B) DCR of total group; (C) CR of total group; (D) PR of total group; (E) SD of total group; (F) ORR of anti-PD1-containing regimens; (G) ORR of second line therapy or beyond; (H) DCR of second line therapy or beyond. ORR, objective response rate; DCR, disease control rate; CR, complete response; PR, partial response; SD, stable disease; PD1, programmed cell death protein 1. [file Image_1.jpeg]

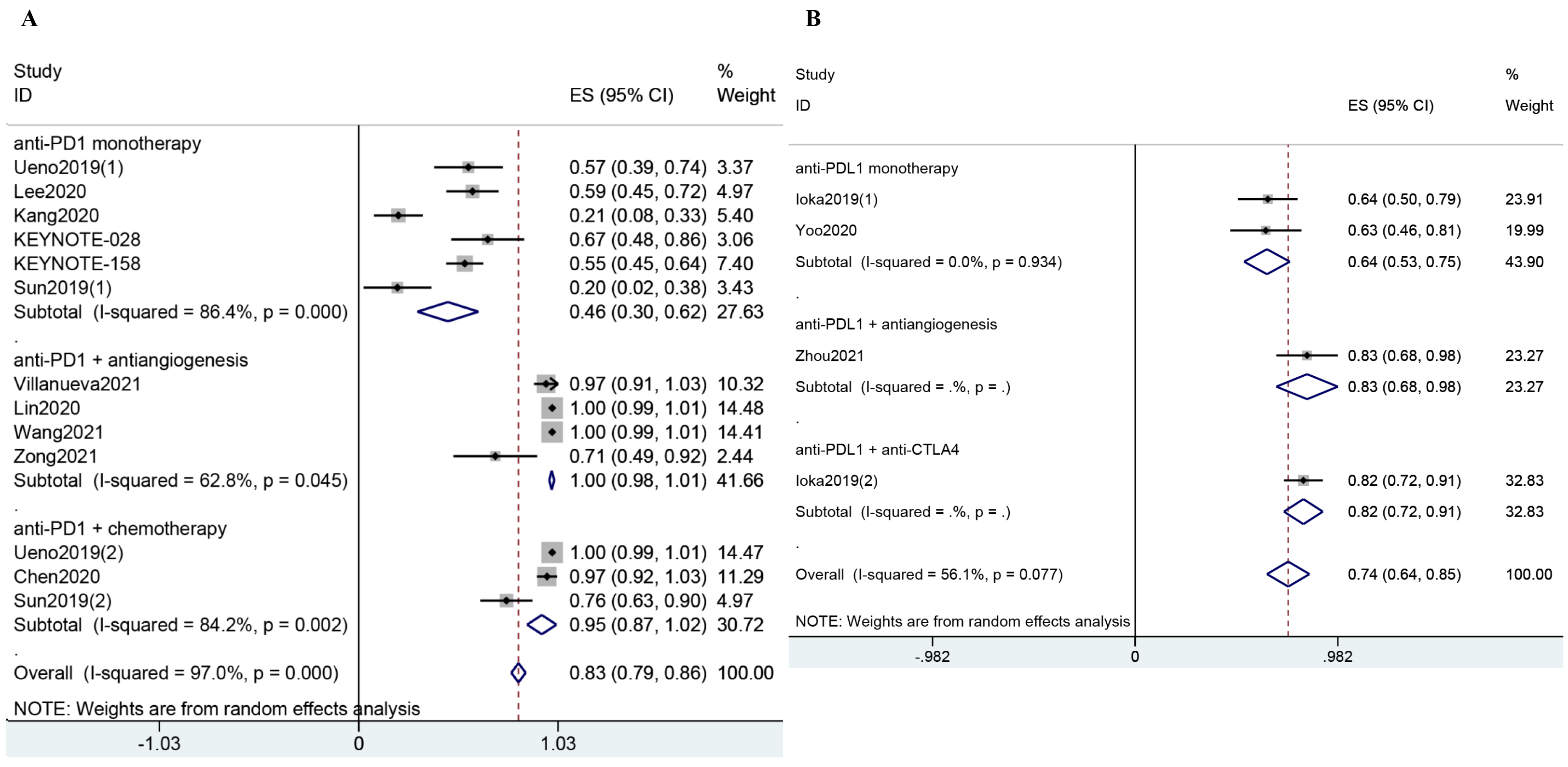

Supplement: Supplementary Figure 2 — Forest plot of any-grade AEs in anti-PD1-containing regimens and anti-PDL1-containing regimens. (A) Anti-PD1-containing regimens; (B) anti-PDL1-containing regimens. Three studies had more than one subgroup of interest. Specifically, patients were allocated to nivolumab group [Ueno2019(1)] or nivolumab/GemCis group [Ueno2019(2)] in Ueno2019 study; PD1 inhibitor monotherapy group [Sun2019(1)] or PD1 inhibitor plus chemotherapy group [Sun2019(2)] in Sun2019 study; durvalumab group [Ioka2019(1)] or durvalumab/tremelimumab group [Ioka2019(2)] in Ioka2019 study. Heterogeneity across studies was evaluated by the Cochran Q chi-square test and I2 statistic, with p<0.1 for the Q test deemed to have high heterogeneity and I2 >50% regarded as an indicator of moderate-to-high heterogeneity. If separate verdicts from the Q test and I2 statistic were at opposite poles, we would give priority to the conclusion from I2 statistic since the former is proverbially underpowered to detect heterogeneity. AEs, adverse events; ES, effect size; CI, confidence interval; PD1, programmed cell death protein 1; PDL1, programmed cell death ligand 1; CTLA4, cytotoxic T lymphocyte antigen 4; GemCis, gemcitabine + cisplatin. [file Image_2.jpeg]
