# Supplementary material for: Efficacy and Safety of Anti-PD1/PDL1 in Advanced Biliary Tract Cancer: A Systematic Review and Meta-Analysis
Source: Front Immunol. 2022 Mar 2;13:801909. doi: 10.3389/fimmu.2022.801909 (PMC8924050; doi:10.3389/fimmu.2022.801909)
Supplement: Supplementary file 4 [file Table_1.docx]

| **SUPPLEMENTARY TABLE 1 \|** Quality assessment of included studies. | | | | | | | |
| --- | --- | --- | --- | --- | --- | --- | --- |
| ROBINS-I | | | | | | | |
| Study | Bias due to  confounding | Bias in selection of participants into the study | Bias in classification of interventions | Bias due to deviations from intended interventions | Bias due to missing data | Bias in measurement of outcomes | Bias in selection of the reported result |
| Kim et al. (2020) | Moderate | low | low | low | low | low | low |
| Kang et al. (2020) | Moderate | low | low | low | low | low | low |
| KEYNOTE-028 | Moderate | low | low | low | low | low | low |
| KEYNOTE-158 | Moderate | low | low | low | low | low | low |
| Yoo et al. (2020) | Moderate | low | low | low | low | low | low |
| Lin et al. (2020) | Moderate | low | low | low | low | low | low |
| Arkenau et al. (2018) | Moderate | low | low | low | low | low | Moderate |
| Wang et al. (2021) | Moderate | low | low | low | low | low | low |
| Klein et al. (2020) | Moderate | low | low | low | low | low | Moderate |
| Chen et al. (2020) | Moderate | low | low | low | low | low | low |
| Ueno et al. (2019) | Moderate | low | low | low | low | low | low |
| ROBINS-I, Risk Of Bias In Non-randomised Studies - of Interventions | | | | | | | |

| The JBI critical appraisal tool for case series | | | | | | | | | | | |
| --- | --- | --- | --- | --- | --- | --- | --- | --- | --- | --- | --- |
| Study | Q1 | Q2 | Q3 | Q4 | Q5 | Q6 | Q7 | Q8 | Q9 | Q10 | Overall appraisal |
| Lee et al. (2020) | Yes | Yes | Yes | Unclear | Unclear | Yes | Yes | Yes | Yes | Yes | Include |
| Sun et al. (2019) | Yes | Yes | Yes | Unclear | Unclear | Yes | Yes | Yes | Yes | Yes | Include |
| Numbers Q1-Q10 in heading signified: Q1, were there clear criteria for inclusion in the case series? Q2, was the condition measured in a standard, reliable way for all participants included in the case series? Q3, were valid methods used for identification of the condition for all participants included in the case series? Q4, did the case series have consecutive inclusion of participants? Q5, did the case series have complete inclusion of participants? Q6, was there clear reporting of the demographics of the participants in the study? Q7, was there clear reporting of clinical information of the participants? Q8, were the outcomes or follow up results of cases clearly reported? Q9, was there clear reporting of the presenting site(s)/clinic(s) demographic information? Q10, was statistical analysis appropriate? | | | | | | | | | | | |

| **SUPPLEMENTARY TABLE 2 \|** Pooled results of common AEs of anti-PD1/PDL1 in aBTC. | | | | | | | | | | |
| --- | --- | --- | --- | --- | --- | --- | --- | --- | --- | --- |
| **AEs** |  | **Any-grade AEs** | | | |  | **Grade 3-4 AEs** | | | |
|  |  | **N** | **ES (95%CI), %** | ***I^2^*, %** | **p** |  | **N** | **ES (95%CI), %** | ***I^2^*, %** | **p** |
| RCCEP |  | 59 | 45.1 (10.9, 79.3) | 87.4 | 0.005 |  | 59 | 0.0 (-0.4, 0.5) | 0.0 | 0.972 |
| Hypertension |  | 135 | 39.9 (29.0, 50.7) | 44.6 | 0.125 |  | 114 | 15.4 (8.8, 22.0) | 0.0 | 0.881 |
| Hypoalbuminemia |  | 59 | 36.0 (20.4, 51.6) | 38.6 | 0.202 |  | 59 | 0.0 (-0.4, 0.5) | 0.0 | 0.972 |
| Leukopenia |  | 182 | 34.0 (2.5, 65.5) | 97.2 | <0.001 |  | 158 | 6.2 (2.2, 10.3) | 94.4 | <0.001 |
| Decreased appetite |  | 191 | 26.2 (11.6, 40.8) | 86.4 | <0.001 |  | 191 | 0.1 (-0.4, 0.7) | 20.5 | 0.279 |
| Asthenia |  | 150 | 25.8 (-0.3, 52.0) | 94.5 | <0.001 |  | 176 | 0.0 (-0.2, 0.2) | 0.0 | 0.789 |
| Abdominal pain |  | 74 | 22.6 (8.1, 37.0) | 59.5 | 0.060 |  | 74 | 0.1 (-0.7, 0.9) | 0.0 | 0.559 |
| Nausea |  | 467 | 20.8 (12.2, 29.4) | 89.6 | <0.001 |  | 382 | 0.1 (-0.2, 0.3) | 0.0 | 0.574 |
| Thrombocytopenia |  | 256 | 18.9 (6.8, 31.1) | 95.0 | <0.001 |  | 241 | 3.5 (0.9, 6.0) | 91.3 | <0.001 |
| Anemia |  | 324 | 18.0 (12.6, 23.5) | 97.3 | <0.001 |  | 231 | 0.2 (-0.6, 1.1) | 78.7 | <0.001 |
| Neutropenia |  | 143 | 17.9 (1.3, 34.6) | 89.8 | <0.001 |  | 119 | 0.2 (-1.0, 1.4) | 65.1 | 0.057 |
| Elevated bilirubin |  | 169 | 17.8 (7.5, 28.1) | 70.6 | 0.009 |  | 91 | 8.9 (-0.4, 18.3) | 58.6 | 0.090 |
| ALP increase |  | 121 | 16.8 (5.1, 28.6) | 70.3 | 0.034 |  | 67 | 0.0 (-0.4, 0.4) | 0.0 | 0.988 |
| AST increase |  | 227 | 16.7 (7.2, 26.1) | 79.5 | <0.001 |  | 173 | 0.1 (-0.4, 0.6) | 3.0 | 0.398 |
| Fever |  | 374 | 14.9 (7.8, 21.9) | 91.8 | <0.001 |  | 374 | 0.0 (-0.1, 0.2) | 0.0 | 0.995 |
| PPE |  | 54 | 14.8 (5.3, 24.2) | 0.0 | 0.838 |  | 54 | 0.1 (-0.8, 1.0) | 0.0 | 0.322 |
| γ-GT increase |  | 67 | 13.3 (-1.3, 27.9) | 70.2 | 0.067 |  | 67 | 9.4 (2.4, 16.3) | 0.0 | 0.344 |
| Fatigue |  | 520 | 12.8 (9.5, 16.1) | 95.9 | <0.001 |  | 456 | 0.0 (-0.2, 0.3) | 21.5 | 0.233 |
| Malaise |  | 118 | 12.4 (-0.7, 25.4) | 84.2 | <0.001 |  | 118 | 0.0 (-0.3, 0.4) | 0.0 | 1.000 |
| Proteinuria |  | 140 | 12.2 (1.3, 23.0) | 87.6 | <0.001 |  | 140 | 0.1 (-0.4, 0.6) | 0.0 | 0.573 |
| Rash |  | 531 | 11.5 (7.3, 15.7) | 87.8 | <0.001 |  | 477 | 0.1 (-0.2, 0.4) | 26.3 | 0.178 |
| Gastrointestinal hemorrhage |  | 54 | 10.9 (2.6, 19.2) | 0.0 | 0.687 |  | 54 | 9.3 (1.5, 17.0) | 0.0 | 0.972 |
| Lymphopenia |  | 146 | 10.1 (-0.6, 20.9) | 87.1 | <0.001 |  | 92 | 0.0 (-0.4, 0.5) | 0.0 | 0.604 |
| Pruritus |  | 307 | 9.4 (6.1, 12.6) | 0.0 | 0.957 |  | 214 | 0.1 (-0.3, 0.4) | 0.0 | 0.924 |
| Vomiting |  | 417 | 9.1 (4.9, 13.2) | 87.6 | <0.001 |  | 363 | 0.0 (-0.1, 0.2) | 0.0 | 0.684 |
| Hypothyroidism |  | 401 | 8.5 (3.6, 13.3) | 82.8 | <0.001 |  | 347 | 0.0 (-0.1, 0.2) | 0.0 | 0.976 |
| Stomatitis |  | 86 | 7.9 (-3.5, 19.4) | 78.5 | 0.010 |  | 86 | 0.0 (-0.4, 0.4) | 0.0 | 1.000 |
| Epistaxis |  | 58 | 7.9 (1.0, 14.9) | 0.0 | 0.486 |  | 58 | 0.0 (-0.4, 0.5) | 0.0 | 0.988 |
| Diarrhea |  | 459 | 7.2 (4.0, 10.5) | 79.9 | <0.001 |  | 374 | 0.0 (-0.1, 0.2) | 0.0 | 0.940 |
| Myalgia |  | 118 | 5.1 (-1.4, 11.7) | 67.0 | 0.028 |  | 118 | 0.0 (-0.3, 0.4) | 0.0 | 1.000 |
| Constipation |  | 225 | 4.9 (0.6, 9.1) | 86.5 | <0.001 |  | 225 | 0.0 (-0.2, 0.2) | 0.0 | 0.535 |
| ALT increase |  | 227 | 4.3 (1.3, 7.3) | 87.4 | <0.001 |  | 173 | 0.1 (-0.3, 0.4) | 0.0 | 0.520 |
| Colitis |  | 148 | 4.0 (-2.1, 10.2) | 44.3 | 0.146 |  | 148 | 0.8 (-0.8, 2.4) | 13.9 | 0.323 |
| Blood creatinine increase |  | 60 | 3.5 (-5.8, 12.8) | 69.7 | 0.069 |  | 60 | 0.0 (-0.4, 0.5) | 0.0 | 1.000 |
| Dysgeusia |  | 60 | 3.5 (-5.8, 12.8) | 69.7 | 0.069 |  | 60 | 0.0 (-0.4, 0.5) | 0.0 | 1.000 |
| Hiccups |  | 60 | 3.5 (-5.8, 12.8) | 69.7 | 0.069 |  | 60 | 0.0 (-0.4. 0.5) | 0.0 | 1.000 |
| Severe skin reaction |  | 128 | 3.1 (0.1, 6.0) | 0.0 | 0.771 |  | 128 | 2.1 (-0.4, 4.6) | 0.0 | 0.601 |
| Pneumonitis |  | 231 | 2.7 (-0.1, 5.5) | 74.2 | 0.002 |  | 231 | 0.6 (-0.5, 1.7) | 26.7 | 0.234 |
| Infusion reaction |  | 258 | 2.1 (-0.3, 4.5) | 60.4 | 0.019 |  | 204 | 0.0 (-0.2, 0.2) | 0.0 | 0.507 |
| Maculopapular rash |  | 218 | 2.1 (-0.6, 4.8) | 57.2 | 0.053 |  | 278 | 0.0 (-0.1, 0.2) | 0.0 | 0.474 |
| Febrile neutropenia |  | 60 | 1.8 (-3.9, 7.5) | 52.6 | 0.146 |  | 60 | 1.8 (-3.9, 7.5) | 52.6 | 0.146 |
| Alopecia |  | 155 | 1.5 (-0.8, 3.8) | 82.2 | <0.001 |  | 155 | 0.0 (-0.3, 0.4) | 0.0 | 0.910 |
| Amylase increase |  | 90 | 1.4 (-1.9, 4.7) | 35.3 | 0.213 |  | 90 | 1.4 (-1.9, 4.7) | 35.3 | 0.213 |
| Peripheral neuropathy |  | 206 | 0.4 (-0.6, 1.4) | 69.9 | 0.005 |  | 206 | 0.0 (-0.2, 0.3) | 0.0 | 0.962 |
| Hepatitis |  | 186 | 0.3 (-0.5, 1.1) | 20.7 | 0.286 |  | 218 | 0.1 (-0.3, 0.5) | 0.0 | 0.758 |
| Myositis |  | 128 | 0.2 (-0.6, 0.9) | 0.0 | 0.378 |  | 128 | 0.2 (-0.6, 0.9) | 0.0 | 0.378 |
| Type 1 diabetes mellitus |  | 128 | 0.2 (-0.6, 0.9) | 0.0 | 0.378 |  | 128 | 0.2 (-0.6, 0.9) | 0.0 | 0.378 |
| Hyperthyroidism |  | 128 | 0.2 (-0.6, 0.9) | 0.0 | 0.378 |  | 160 | 0.1 (-0.5, 0.6) | 39.5 | 0.191 |
| Thyroiditis |  | 128 | 0.2 (-0.6, 0.9) | 0.0 | 0.378 |  | 128 | 0.0 (-0.2, 0.2) | 0.0 | 0.940 |
| Anaphylactic reaction |  | 60 | 0.1 (-0.6, 0.8) | 0.4 | 0.316 |  | 60 | 0.1 (-0.6, 0.8) | 0.4 | 0.316 |
| Myocarditis |  | 60 | 0.1 (-0.6, 0.8) | 0.4 | 0.316 |  | 60 | 0.1 (-0.6, 0.8) | 0.4 | 0.316 |
| Oedema |  | 239 | 0.1 (-0.5, 0.7) | 38.5 | 0.165 |  | 239 | 0.0 (-0.1, 0.2) | 0.0 | 1.000 |
| AEs, adverse events; PD1, programmed cell death protein 1; PDL1, programmed cell death ligand 1; aBTC, advanced biliary tract cancer; ES, effect size; CI, confidence interval; RCCEP, reactive cutaneous capillary endothelial proliferation; ALP, alkaline phosphatase; AST, aspartate aminotransferase; PPE, Palmar-Plantar Erythrodysesthesia syndrome; γ-GT, gamma-glutamyltransferase; ALT, alanine aminotransferase.  Heterogeneity across studies was evaluated by the Cochran *Q* chi-square test and *I^2^* statistic, with p<0.1 for the *Q* test deemed to have high heterogeneity and *I^2^*>50 % regarded as an indicator of moderate-to-high heterogeneity. If separate verdicts from the *Q* test and *I^2^* statistic were at opposite poles, we would give priority to the conclusion from *I^2^* statistic since the former is proverbially underpowered to detect heterogeneity. | | | | | | | | | | |

| **SUPPLEMENTARY TABLE 3 \|** Pooled results after omitting the studies that influenced the robustness of pooled any-grade AEs. | | | | |
| --- | --- | --- | --- | --- |
| **total group** | |  | **anti-PD1/PDL1 + antiangiogenesis group** | |
| **Study omitted** | **pooled any-grade AEs** |  | **Study omitted** | **pooled any-grade AEs** |
| Sun2019(1) | 85.6% (95%CI 82.4% to 88.7%) |  | Villanueva2021 | 93.2% (95%CI 85.3% to 101.1%) |
| Yoo2020 | 74.4% (95%CI 69.5% to 79.3%) |  | Wang2021 | 93.2% (95%CI 85.3% to 101.1%) |
| Zong2021 | 74.5% (95%CI 69.7% to 79.4%) |  |  |  |
| Ioka2019(2) | 75.9% (95%CI 71.6% to 80.2%) |  |  |  |
|  |  |  |  |  |
| **anti-PD1 + antiangiogenesis group** | |  | **second line therapy or beyond** | |
| **Study omitted** | **pooled any-grade AEs** |  | **Study omitted** | **pooled any-grade AEs** |
| Villanueva2021 | 95.8% (95%CI 88.3% to 103.4%) |  | KEYNOTE-158 | 77.3% (95%CI 72.7% to 82.0%) |
| Wang2021 | 95.9% (95%CI 88.3% to 103.4%) |  | Ioka2019(1) | 65.7% (95%CI 51.7% to 79.7%) |
|  |  |  | Zong2021 | 65.7% (95%CI 51.7% to 79.7%) |
| AEs, adverse events; PD1, programmed cell death protein 1; PDL1, programmed cell death ligand 1.  Note: Two studies had more than one subgroup of interest. Specifically, patients were allocated to PD1 inhibitor monotherapy group (Sun2019[1]) or PD1 inhibitor plus chemotherapy group (Sun2019[2]) in Sun2019 study; durvalumab group (Ioka2019[1]) or durvalumab/tremelimumab group (Ioka2019[2]) in Ioka2019 study. | | | | |
